# Averting climate change’s health effects in Fiji

**DOI:** 10.2471/BLT.15.021115

**Published:** 2015-11-01

## Abstract

Pacific islanders face up to the dire health effects of global warming. Atasa Moceituba and Monique Tsang report.

Kaushik Sindhu Lal learned the hard way. After visiting a friend on the edge of the Fijian capital of Suva for a few days last year the 24-year-old came home shivering and sweating, his head and body aching.

“There were lots of bushes and mosquitoes out there,” Lal says. “We were playing football and wearing shorts. I didn’t think of using insect repellent,” he says.

Lal is one of some 27 000 Fijians to have had dengue fever in 2013–14, in one of the Pacific island’s largest known outbreaks of the viral mosquito-borne infection.

“Fiji and other tropical island states in the Pacific are particularly susceptible to the effects of climate change, especially rising sea levels, because they are made up of many small islands surrounded by fragile coral reef ecosystems,” says Dr Rokho Kim, an environmental health specialist with the World Health Organization (WHO) Regional Office for the Western Pacific based in Fiji.

“The industrialized countries are mainly responsible for the carbon dioxide emissions that cause global warming,” Kim says. “And small developing countries like Fiji and other Pacific island nations are suffering.

“They are some of the most vulnerable places on the planet to the health effects of climate change,” he says.

The inhabitants of many small island states around the world are suffering the effects of climate-sensitive health problems related to extreme weather, such as tropical cyclones, storm surges, flooding and drought, according to the 2014 Intergovernmental Panel on Climate Change’s *Fifth assessment report*.

The incidence of infectious diseases in some of these areas, such as dengue and malaria, is increasing, while health problems linked to the deterioration in the quality and quantity of freshwater are prevalent, the report says.

Climate change can exacerbate already difficult circumstances, as the increased disease burden results in overstretched health-care services and a greater risk of disease and death among vulnerable groups, especially young children, women of reproductive age, older people and people with disabilities.

Dengue occurs most during the wet season in the tropics.

Scientists studying the 2013–14 outbreak believe climate change was one factor but that increasingly mobile populations, urbanization and the appearance of a virus serotype that had not been seen before in Fiji also contributed to its severity.

“The outbreak shows how the health effects of climate change can be dire in less developed countries where public hygiene is inadequate and health systems are weak,” Kim says.

“The initial hotspots of Fiji’s 2013–14 dengue outbreak were in the squatter settlements and poor neighbourhoods in and around Suva,” Kim adds.

It is one of several large outbreaks in the Pacific that WHO and its partners investigated in recent years.

They found that large outbreaks often follow extreme weather events, for example, a drought-induced outbreak of diarrhoeal diseases in Tuvalu in 2011, a post-flood leptospirosis outbreak in Fiji in 2012; and floods in 2014 that triggered an epidemic of diarrhoeal disease in the Solomon Islands.

“We have plenty of evidence to show what is happening. Anyone who is still unsure should come to the Pacific and see for themselves,” says Kim.

World leaders gather later this month in Paris, France at the United Nations Climate Change Summit from 30 November to 11 December to hammer out an agreement to reduce carbon dioxide emissions and to consider proposals to support developing countries adapt to the effects of climate change.

“We hope that the world leaders will pay attention to what is happening here and take immediate action.”

Rokho Kim

“We hope that the world leaders will pay attention to what is happening here and take immediate action. That will be good news not only for Fijians and other Pacific islanders, but also for coming generations all over the world,” Kim says.

Dengue is one of several infectious tropical diseases, including typhoid fever, leptospirosis, zika and chikungunya, which are endemic to the Pacific islands and on the increase, adding to the already heavy toll of noncommunicable diseases (NCDs).

The prevalence of hypertension, diabetes, obesity and other NCDs is so high that health ministers from 19 Pacific countries and areas declared in 2011 that the “Blue Continent” was facing an “NCD crisis”.

By the time the dengue outbreak struck in late 2013, “Fiji’s health system was already overwhelmed by the burden of other diseases,” Kim says.

Stepping up the response to disease outbreaks has been a major part of efforts to protect the health of the 900 000 strong population of Fiji, an archipelago of more than 300 islands.

“One of WHO’s priorities in the Pacific islands is to help build and reinforce their health systems,” Kim says. “We are working with 13 countries to implement national climate change and health action plans that we helped to develop.”

The action plans are part of a global WHO strategy to help countries make their health systems more resilient to the effects of climate change.

For Dr Meciusela Tuicakau from Fiji’s Ministry of Health and Medical Services, the dengue outbreak of 2013–14 exposed weaknesses in the country’s ability to detect outbreaks early enough to warn communities and take measures.

“Under our paper-based system for reporting cases, patients’ addresses were not recorded and that delayed timely measures to protect the population,” Tuicakau says.

“We couldn’t intervene when we should have because of the limited information received,” he says.

Fiji’s health ministry has since revised the paper form to include a field for the patient’s address and phone number, and now it is developing a web-based reporting system for the rapid sharing of information about disease outbreaks to improve its response in future – a system that will be piloted soon.

“The new system will enable the use of tablets and mobile phones for the input, storage and sharing of case information,” Tuicakau says.

“It will be used by doctors and nurses when they assess patients that arrive at hospitals, laboratory workers who analyse blood and tissue samples and environmental health officers who visit communities to take preventive action and monitor the situation,” he says.

A key challenge for reducing the transmission of infectious diseases is changing people’s behaviour to improve hygiene and reduce their contact with mosquitoes.

Dengue is not transmitted directly from person to person but by the bite of a mosquito infected with the virus.

It is prevented by reducing mosquito habitats, such as the removal of standing water, and avoiding mosquito bites with nets, suitable clothing and insect repellent or by avoiding the outdoors at dusk and dawn, when mosquitoes are most active.

“In Fiji, for example, coconut shells and abandoned car tyres are well known breeding grounds for mosquitoes – so these need to be disposed of properly,” says Tuicakau.

“Some people sleep outside when it’s hot and humid, without using mosquito nets or insect repellent, exposing themselves to the risk of dengue and other mosquito-borne diseases,” he says.

Helping to change these kinds of behaviour is just what Alita Goneva and the other Climate Champions of the Fiji Red Cross Society set out to do.

Since September 2012, the Fiji Red Cross Society has been collaborating with the health ministry by training volunteers to educate people in their communities about health protection.

For instance, they learn about the importance of regular hand washing, boiling drinking water and the hygienic handling of food to prevent diarrhoeal and other diseases.

“We assist them by conducting clean-up campaigns in their communities, such as destroying mosquitoes’ breeding grounds and rubbish separation.

“We also explain the need to use protective wear when out on the farm, for example, to prevent leptospirosis,” says Goneva, referring to a climate-sensitive disease caused by bacteria carried by rodents, dogs, cattle and other mammals.

Goneva and other Fiji Red Cross Society trainers then accompany the volunteers when they go back to their neighbourhoods to engage fellow community members in these practices.

“People need to be made aware of climate change as it often exacerbates a disease problem that already exists in the community, like dengue.”

Kelera Oli

“People need to be made aware of climate change as it often exacerbates a disease problem that already exists in the community, like dengue,” says Kelera Oli, the climate change and health officer at Fiji’s health ministry.

**Figure:**
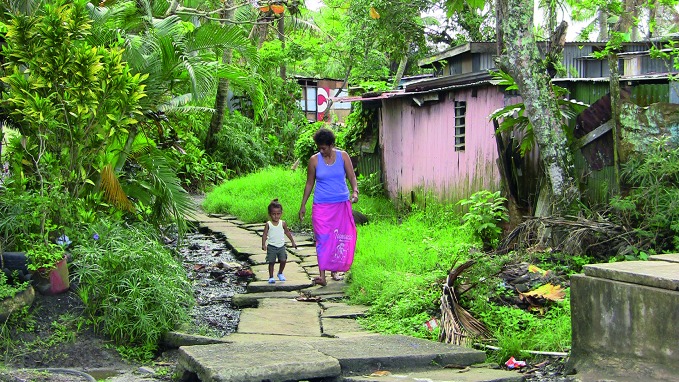
Residents of Wailea, a settlement with extremely poor sanitary conditions outside the Fijian capital of Suva.

WHO

**Figure:**
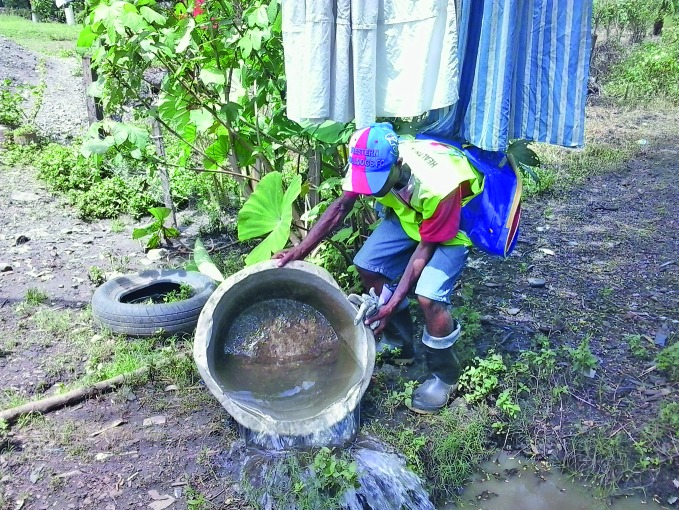
Fiji Red Cross Society volunteer clears water from an abandoned car tyre to destroy a potential mosquito breeding ground.

Fiji Red Cross Society

